# Pregnancy-associated diamine oxidase originates from extravillous trophoblasts and is decreased in early-onset preeclampsia

**DOI:** 10.1038/s41598-018-24652-0

**Published:** 2018-04-20

**Authors:** Philipp Velicky, Karin Windsperger, Karin Petroczi, Sophie Pils, Birgit Reiter, Tamara Weiss, Sigrid Vondra, Robin Ristl, Sabine Dekan, Christian Fiala, David E. Cantonwine, Thomas F. McElrath, Bernd Jilma, Martin Knöfler, Thomas Boehm, Jürgen Pollheimer

**Affiliations:** 1 Department of Obstetrics and Gynaecology, Reproductive Biology Unit, Medical University of Vienna, Vienna, Austria; 2 Department of Clinical Pharmacology, Medical University of Vienna, Vienna, Austria; 3 Department of Obstetrics and Gynaecology, Division of General Gynaecology and Gynaecologic Oncology, Medical University of Vienna, Vienna, Austria; 4 Forensic Toxicology, Clinical Institute of Laboratory Medicine, Medical University of Vienna, Vienna, Austria; 5 Children’s Cancer Research Institute, Vienna, Austria; 6 Section for Medical Statistics (IMS), Center of Medical Statistics, Informatics and Intelligent Systems, Medical University of Vienna, Vienna, Austria; 7 Clinical Institute of Pathology, Medical University of Vienna, Vienna, Austria; 8 Gynmed Clinic, Vienna, Austria; 9 Division of Maternal and Fetal Medicine, Brigham and Women’s Hospital, Harvard Medical School, Boston, MA United States

## Abstract

Human extravillous trophoblast (EVT) invasion of the pregnant uterus constitutes a pivotal event for the establishment of the maternal-fetal interface. Compromised EVT function manifesting in inadequate arterial remodeling is associated with the severe pregnancy disorder early-onset preeclampsia (eoPE). Recent studies suggest that EVTs invade the entire uterine vasculature including arteries, veins and lymphatics in the first trimester of pregnancy. We therefore hypothesized that EVT-derived factors accumulate in the circulation of pregnant women early in gestation and may serve to predict eoPE. In contrast to published literature, we demonstrate that placenta-associated diamine oxidase (DAO) is not expressed by maternal decidual cells but solely by EVTs, especially when in close proximity to decidual vessels. Cultures of primary EVTs express and secret large amounts of bioactive DAO. ELISA measurements indicate a pregnancy-specific rise in maternal DAO plasma levels around gestational week (GW) 7 coinciding with vascular invasion of EVTs. Strikingly, DAO levels from eoPE cases were significantly lower (40%) compared to controls in the first trimester of pregnancy but revealed no difference at mid gestation. Furthermore, DAO-containing pregnancy plasma rapidly inactivates pathophysiologically relevant histamine levels. This study represents the first proof of concept suggesting EVT-specific signatures as diagnostic targets for the prediction of eoPE.

## Introduction

During early human placental development distinct epithelial villous cytotrophoblast (vCTB) progenitors follow two alternative differentiation pathways. Villous CTBs can fuse to form a multinucleated, postmitotic syncytium, which secretes pregnancy-maintaining hormones and controls nutrient transport and gas exchange^1^. However, upon attachment to the pregnant uterus (decidua) CTB progenitors at the proximal end of placental cell columns give rise to so-called extravillous trophoblasts (EVTs)^2^. This trophoblast subtype gains an invasive, mesenchymal phenotype and infiltrates the maternal uterus as deep as the inner third of the myometrium^3^. EVTs colonize uterine spiral arteries to modulate the uteroplacental blood flow and were shown, by executing a program of vascular mimicry, to convert spiral arteries from narrow vessels with high blood pressure to wider, low pressure conduits^4^. In addition, recent findings demonstrate that EVTs also invade uterine veins and lymphatics^5^. The process of EVT invasion and arterial remodeling seems disturbed in a variety of pregnancy diseases including preeclampsia (PE)^6,7^. Preeclampsia, affecting 3% to 8% of pregnancies, is diagnosed by the de novo development of high blood pressure, non-dependent edema and proteinuria and comprises the second largest cause of maternal deaths^8^. Preeclampsia is clinically subdivided into an early and late onset variant, the former classified by delivery before gestational week (GW) 34 and associated with a 10-fold higher risk of mortality^9^. Early-onset PE (eoPE) is clearly associated with incomplete spiral artery remodeling and related changes in uteroplacental blood flow as well as intrauterine growth restriction (IUGR)^10,11^.

The enzymatic activity of diamine oxidase (DAO, encoded by *AOC1*) is near the detection limit in serum or plasma of non-pregnant individuals but increases several hundred-fold during gestation^12^. The increase in activity has been confirmed by ELISA measurements of DAO antigen concentrations in sera and plasma of pregnant women^13^. DAO oxidizes polyamines including putrescine and spermidine and is the only extracellular enzyme capable of inactivating histamine in humans^13,14^. DAO shows the highest expression in intestine, kidney and placenta^15^. Although some evidence exists to suggest DAO mRNA production in placental trophoblasts^16^, pregnancy-associated DAO production and activity was generally assigned to maternal decidual stroma cells^17–19^. Human placenta tissue has previously demonstrated high histamine degrading capacity and placental tissue has been used to purify DAO to homogeneity^20^. Low DAO activity in the first trimester of pregnancy has been associated with an increased risk of fetal death by about sixteen fold, suggesting a pivotal role for DAO during human pregnancy^21^.

The overall aim of this study was to evaluate whether EVT-secreted factors appear in the circulation of pregnant women early in gestation and whether these factors can serve as predictive markers for eoPE. Therefore, we investigated the uteroplacental expression pattern of DAO, its capacity to inactivate histamine in the serum of pregnant women and its potential as biomarker for eoPE.

## Results

### Placenta-associated DAO specifically localizes to EVTs

**Figure 1:**
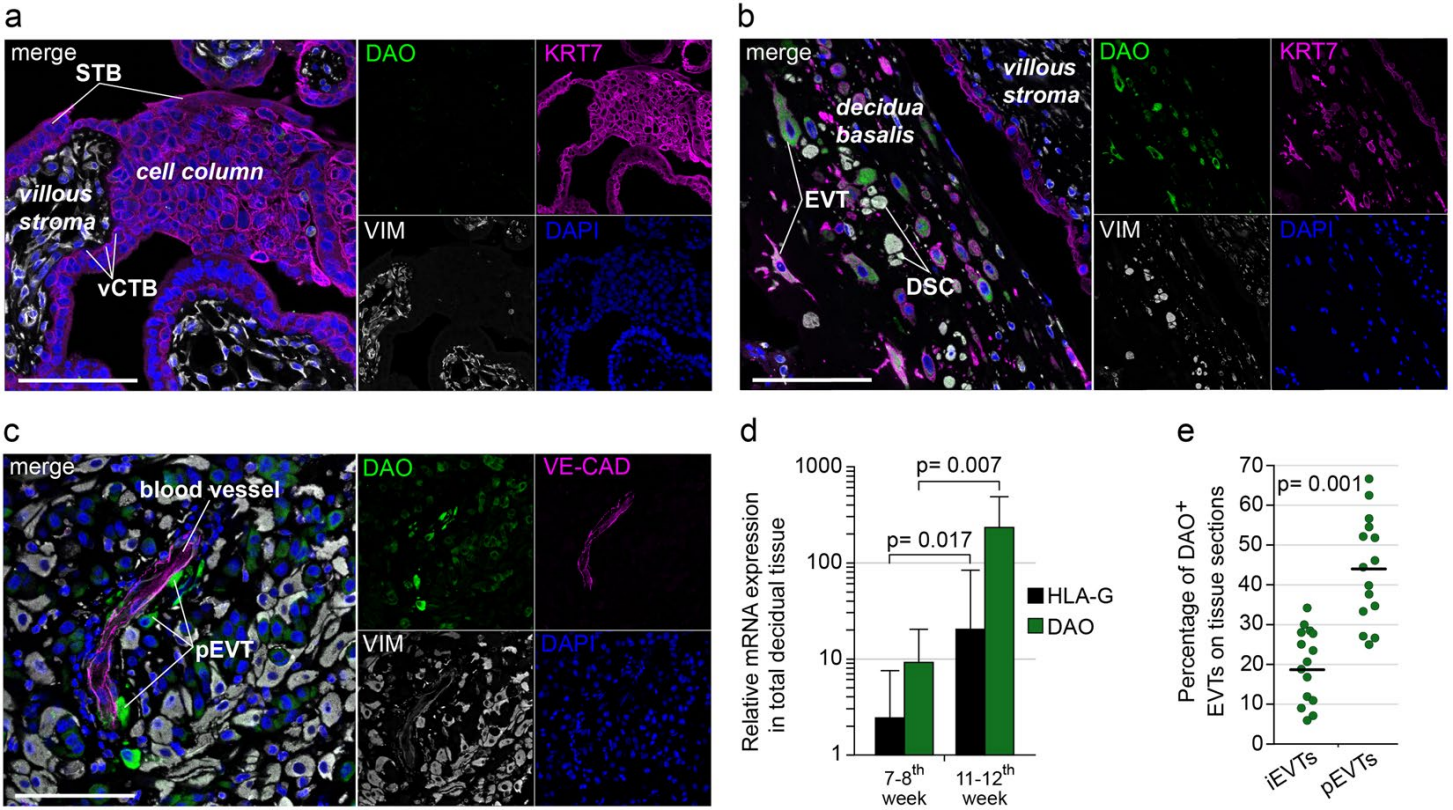
DAO is exclusively expressed by EVTs at the maternal-fetal interface (**a**,**b** and **c**) IF stainings of (**a**) placental villous, (**b**) decidua basalis tissue sections (n = 6, GW 7–12) showing DAO (green), keratin 7 (KRT7, magenta) and vimentin (VIM, grey). (**c**) Staining of a decidua basalis tissue section (n = 5, GW 7–12) showing DAO (green), VE-cadherin (VE-CAD, magenta), VIM (grey). DAPI (blue) was used to visualize nuclei. (Scale bars, 100 µm). (**d**) QPCR analysis of DAO and HLA-G mRNA expression in lysates from decidua basalis tissues. Each bar represents the mean (±SD, n = 8). (**e**) Quantification of DAO + interstitial (**i**) or perivascular (**p**) EVTs relative to the total number of EVTs. Each dot represent the percentage of DAO + EVTs per microscopic field (n = 5; 8–10 microscopic images per tissue). The black bars show mean values (GW 8–11).

Genome-wide RNA-sequencing expression profiles across human tissues retrieved from the Human Protein Atlas portal (www.proteinatlas.org) and a scientific report^22^ revealed high DAO expression levels in colon, intestine, kidneys and the placenta (Supplementary Fig. S1). Subsequently, we performed immunofluorescence (IF) triple-stainings in sections of human placental and decidual tissues. Placental stromal cells, vCTBs, syncytiotrophoblasts (STBs) and cell column trophoblasts of placental villi were consistently negative for DAO (Fig. 1a). In contrast, we found intense DAO expression in trophoblasts after detachment from placental cell columns and subsequent invasion into decidual tissue (Supplementary Fig. S2a and S2b). IF staining of decidua basalis tissue sections confirmed strong DAO staining in many but not all EVTs (Fig. 1b and c, Supplementary Fig. S2a and S2b). DAO^+^ EVTs were also found in myometrial tissue sections from hysterectomy specimens at term (Supplementary Fig. S2c). No specific signal was detected in decidual cells of maternal origin including VIM^+^ decidual stromal cells (DSCs), KRT7^+^/HLA-G^−^ glandular cells or CD45^+^ leukocytes (Fig. 1b and c, Supplementary Fig. S2d-S2f). Transcript levels of DAO correlated with increasing levels of human leukocyte antigen G in lysates from mid to late first trimester decidua basalis tissues (Fig. 1d). Of note, DAO is preferentially expressed by EVTs in close proximity to decidual blood vessels (Fig. 1c and e). While the enzyme is found in approximately 20% of interstitial EVTs, it appears to be expressed in almost 45% of EVTs that contact and/or invade the decidual vasculature (Fig. 1e).

### EVTs secrete functionally active DAO

**Figure 2:**
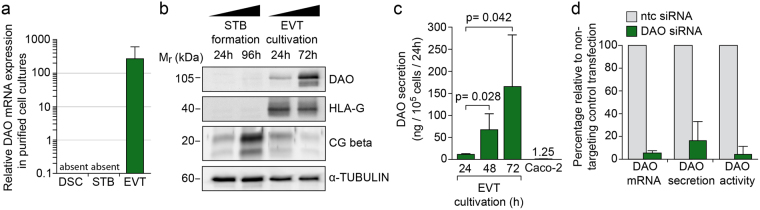
Isolated primary EVTs express and secrete bioactive DAO. (**a**) QPCR analysis of DAO mRNA expression in isolated human DSCs, differentiated STBs and EVTs after 72 hours of cultivation (n = 6, GW 7–12). Bar graphs denote mean values ± SD. (**b**) Western blot illustrating DAO, HLA-G and chorionic gonadotropin beta (CG beta) in differentiating STB and EVT cultures. α-tubulin was used as loading control (n = 3; GW 7–12). (**c**) ELISA quantification of secreted DAO. Each bar represents the mean value (n = 3) ± SD. (**d**) Transfection of primary trophoblasts with siRNAs against DAO or non-targeting control (ntc). QPCR analysis and ELISA were performed to determine DAO mRNA expression and secretion after 48 hours of cultivation (n = 3, GW 7–12), respectively. DAO activity was determined by measuring H_2_O_2_ generation during oxidation of putrescine (n = 3, GW 7–12). Bar graphs display relative mean values (±SD) in response to siRNA-mediated knock-down of DAO (green) compared to control (grey). Uncropped images of western blots can be found in Supplementary Fig. S6.

To investigate DAO secretion in primary trophoblast cultures, we used a recently developed in-house ELISA, since the performance of two commercially available ELISAs were not satisfactory^13^ (also see Material and Methods). First, qPCR and western blot analyzes confirmed lack of DAO in purified cultures of primary DSCs and STB cells, respectively but showed strong expression in primary EVT cultures (Fig. 2a and b, Supplementary Fig. S3a). DAO antigen concentrations in supernatants of cultivated EVTs increased to a similar extent as DAO mRNA and intracellular protein expression during *in vitro* differentiation (Supplementary Fig. S3b and S3c). After three days in culture approximately 10^5^ trophoblasts secreted a mean of 165 ng DAO per ml/24 h or 132-fold more DAO per ml/24 h than Caco-2 cells, a stable human colon carcinoma cell line reported to express DAO^23^ (Fig. 2c). siRNA-mediated knock-down of DAO mRNA strongly diminished DAO expression and secretion (Fig. 2d and Supplementary Fig. S3d and S3e). In addition, a luminescence-based activity assay demonstrated that EVT-derived supernatants efficiently degrade putrescine in a DAO-dependent manner (Fig. 2d and Supplementary Fig. S3f). Presence of DAO-targeting siRNA did not alter the invasive capacity of isolated EVTs (Supplementary Fig. S3g).

### Early detection of DAO in the plasma of pregnant women coincides with invasion of DAO-positive EVTs into arteries and veins

**Figure 3:**
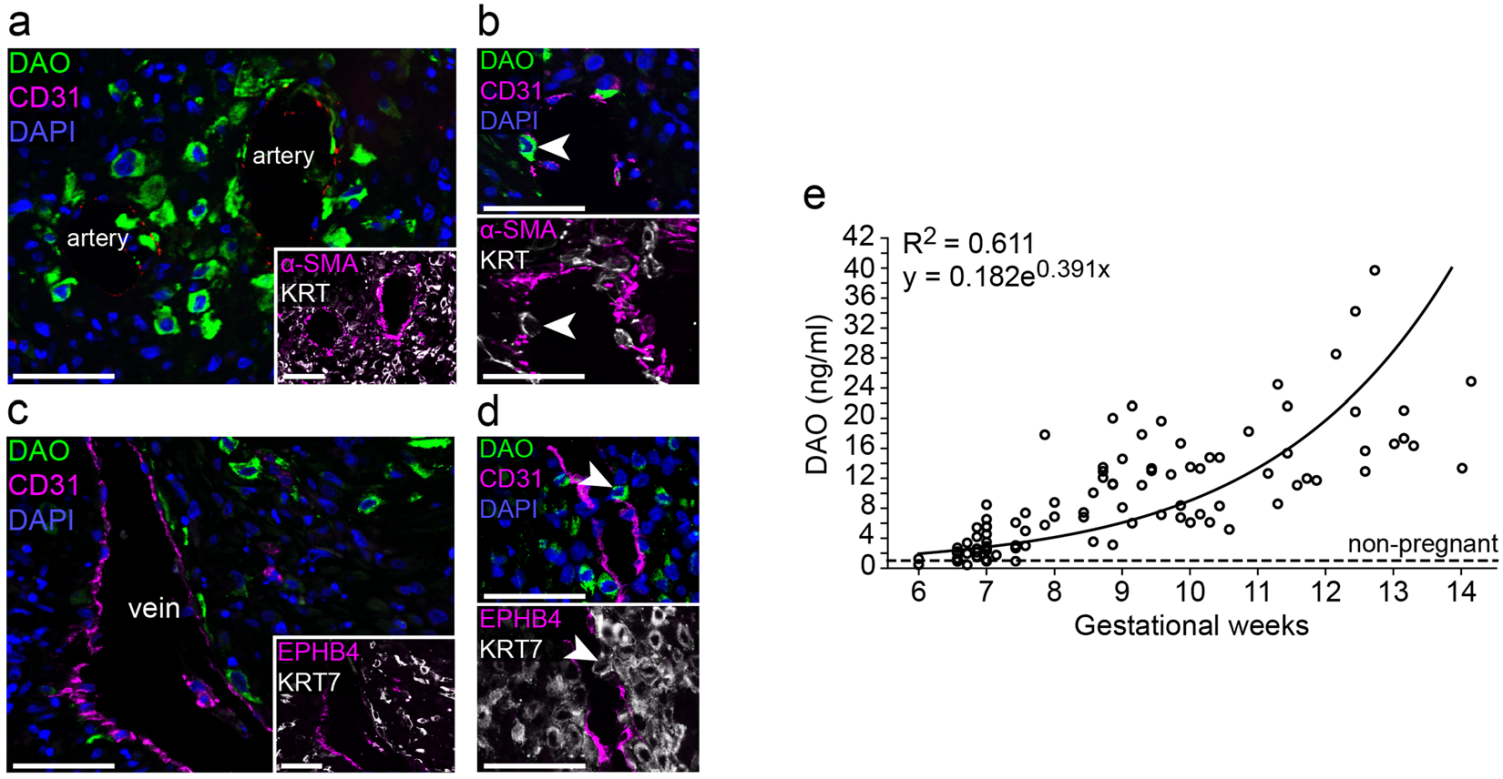
DAO is expressed by EVTs that contact and invade arterial and venous vessels and is appearing at GW 7 in early pregnancy. (**a**) Stainings of decidua basalis tissue sections (n = 4, GW 7–10) showing DAO (green), CD31 (magenta) and DAPI (blue). Inset shows a staining of a serial section for αSMA (magenta) and keratin 7 (KRT7, grey), (n = 4). (**b**) Stainings of decidua basalis tissue serial sections (n = 4, GW 7–10). The upper image shows DAO (green), CD31 (magenta) and DAPI (blue); the lower image αSMA (magenta) and keratin wide-spectrum (KRT, grey). Arrow heads indicate an EVT integrated in the arterial endothelium. (**c**) Stainings of decidua basal tissue sections (n = 4, GW 7–10) of DAO (green), CD31 (magenta) and DAPI (blue). Inset shows a staining of a serial section for ephrin type-B receptor 4 (EPHB4, magenta) and KRT7 (grey). (**d**) Stainings of decidua basalis tissue serial sections (n = 4, GW 7–10). The upper image shows DAO (green), CD31 (magenta) and DAPI (blue); and the lower image EPHB4 (magenta) and KRT7 (grey). Arrow heads indicate an EVT integrated in the venous endothelial layer. (Scale bars, 50 µm) (**e**) ELISA quantification of DAO concentrations in maternal EDTA plasma during early pregnancy (GW 6–14). Circles represent the mean of duplicates (n = 100). Plasma DAO concentrations from 32 non-pregnant healthy subjects are shown as continuous line. The mean DAO concentration in non-pregnant individuals using values only above the Limit of Blank (LOB = 0.27 ng/ml) was 1.2 ng/ml (S.E.M = 0.16).

DAO^+^ EVTs were located around decidual arteries characterized by thick smooth muscle alpha-positive muscular walls as well as in close vicinity to ephrin type-B receptor 4-positive veins (Fig. 3a and c, Supplementary Fig. S4a and S4b). Strong DAO expression was seen in EVTs surrounding decidual arteries at early and late stages of trophoblast-mediated remodeling (Supplementary Fig. 4c). DAO^+^ EVTs also invade and populate the vascular endothelium of venous and arterial vessels and are therefore in direct contact with the maternal blood stream (Fig. 3b and d). Furthermore, we determined DAO plasma antigen levels from early pregnancies reaching a level between 10 and 40 ng/ml by the end of the first trimester (Fig. 3e). More relevant, there is a significant 2.6-fold increase (p < 0.001) in mean DAO concentrations comparing 11 samples from GW 6.0–6.6 (mean = 1.3 ng/ml; S.E.M = 0.4) with 19 samples from GW 6.9–7.0 (mean = 3.42 ng/ml; S.E.M = 0.4), demonstrating that pregnancy-associated DAO appears around GW 7.

### DAO-containing plasma of pregnant women efficiently degrades histamine

**Figure 4:**
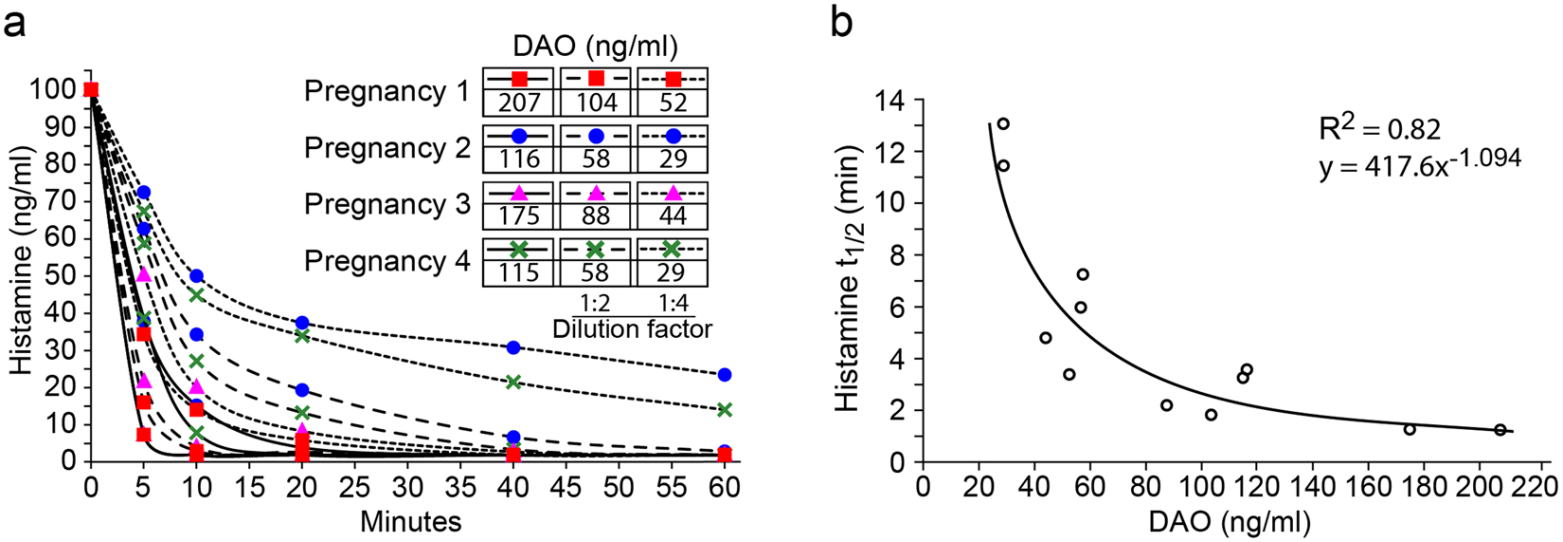
DAO concentrations in maternal serum rapidly degrade histamine to harmless levels. (**a**) Degradation rate of histamine in sera of 4 women in the third trimester of pregnancy was measured. Serum samples were tested undiluted and in 1:2 (50%) and 1:4 (25%) dilutions. (**b**) The histamine half-lives were determined using the data depicted in (A) and plotted against DAO concentrations on the x-axis.

DAO-containing serum of pregnant women rapidly and in a concentration-dependent manner lowered histamine concentrations to levels below 1 ng/ml (Fig. 4a), a histamine concentration with no clinically relevant symptoms^24^. The *in vitro* half-life of histamine decreased to less than 2 minutes at DAO concentrations of 200 ng/ml (Fig. 4b). At DAO concentrations below 50 ng/ml the degradation rate of histamine decreased significantly and its half-life increased exponentially (Fig. 4a and b).

### First trimester DAO plasma levels are reduced in early-onset PE

**Figure 5:**
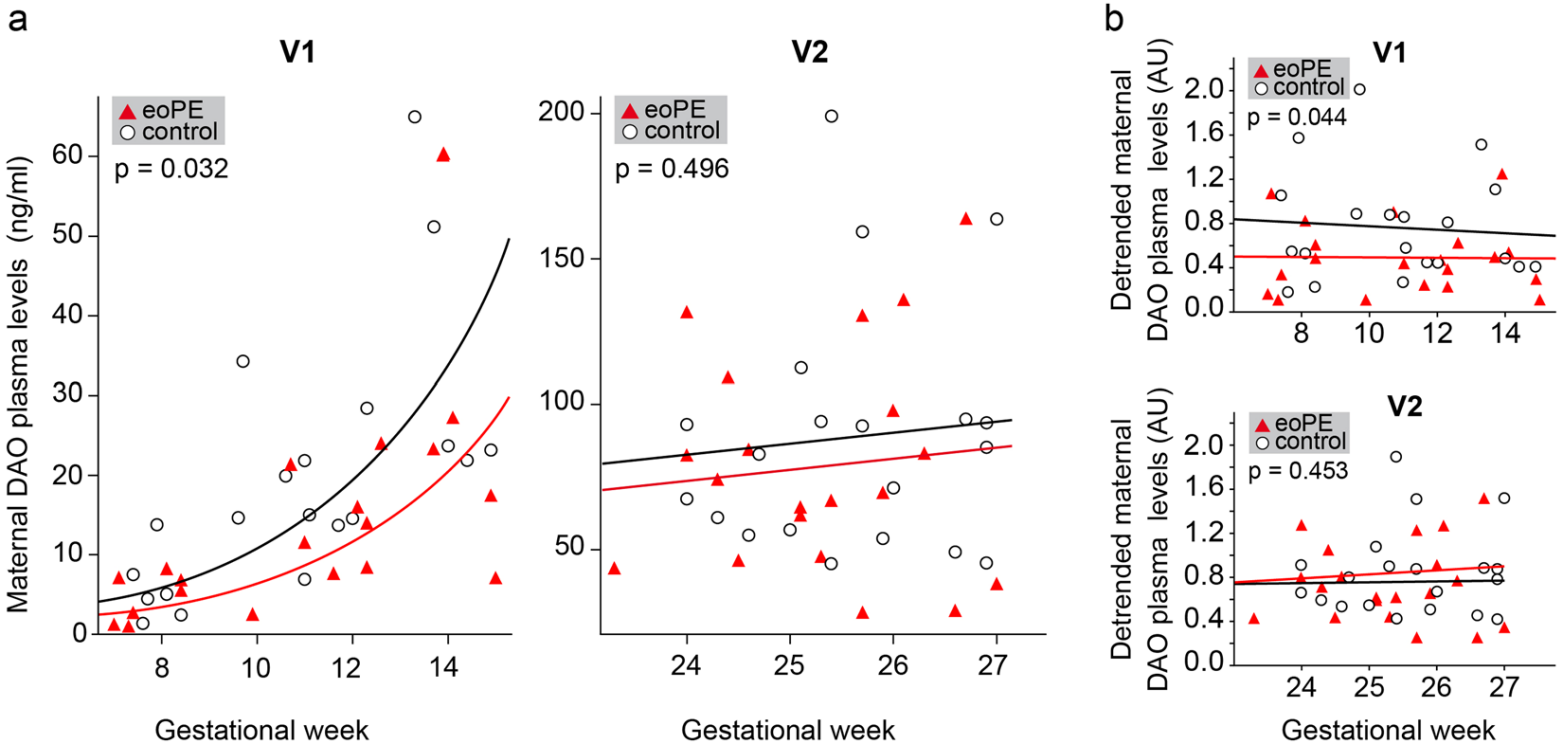
DAO levels in maternal plasma are significantly decreased in eoPE cases during GW 7–15. (**a**) DAO concentrations in maternal EDTA plasma from 20 eoPE and 20 matched control samples at GW 7–15 (V1) and GW 23–27 (V2) are shown. At V1 the median ratio of eoPE to control DAO concentrations is 0.60 (95% confidence intervals (CIs) 0.38, 0.95). (**b**) DAO concentrations from (A) were normalized using a mean DAO activity curve derived from 5 published activity curves using 4PL non-linear regression (Figure S5). The normalized mean DAO values (arbitrary units) at V1 are 0.77 (95% CI 0.55, 0.98) for controls and 0.49 (95% CI 0.31, 0.64) for eoPE subjects; p = 0.044. The mean normalized values at V2 are 0.85 (95% CIs 0.68, 1.03) for controls and 0.76 (95% CI 0.59, 0.92) for eoPE subjects; p = 0.453. The linear regression lines for control (black) and eoPE (red) cases are shown.

DAO plasma concentrations were analyzed in a prospectively collected cohort^25^ including blood samples at GW 7–14 (Visit 1) and GW 23–27 (Visit 2) from uncomplicated pregnancies (n = 20) and women who later on developed eoPE (n = 20). Information on the two sample populations are shown in Table 1. As expected we observed a significant rise of DAO concentrations with gestational age at Visit 1 but no further increase within the time frame of Visit 2 (Fig. 5a). To account for the rising DAO levels a log-linear regression model was used to calculate potential DAO antigen differences between eoPE and control cases. Strikingly, DAO concentrations from eoPE cases were significantly reduced by 40% or showed a median 1.7-fold difference (p = 0.032) at Visit 1 compared to matched healthy control samples (Fig. 5a). No difference (p = 0.496) was measured comparing plasma samples of the same subjects at Visit 2 (Fig. 5a).

**Table 1:** Summary of patient cohort information.

Characteristics	Total (n = 40) Mean (SD) or n (%)	eoPE (n = 20) Mean (SD) or n (%)	No PE (n = 20) Mean (SD) or n (%)	p-value^a^
Age (years)	32.8 (5.5)	32.9 (5.7)	32.6 (5.5)	0.61
BMI at initial visit (kg/m^2^)	30.0 (6.9)	31.0 (7.2)	28.9 (6.5)	0.32
Race				
White	16 (40.0%)	9 (45.0%)	7 (35.0%)	0.99
African-American	12 (30.0%)	5 (25.0%)	7 (35.0%)	
Asian	0 (0.0%)	0 (0.0%)	0 (0.0%)	
Hispanic	8 (20.0%)	3 (15.0%)	5 (25.0%)	
Other	4 (10.0%)	3 (15.0%)	1 (5.0%)	
Nulliparous	9 (22.5%)	3 (15.0%)	6 (30.0%)	0.45
Smoked during pregnancy	5 (12.5%)	4 (20.0%)	1 (5.0%)	0.34
Use of assisted reproductive technology	1 (2.5%)	1 (5.0%)	0 (0.0%)	0.99
Current diagnosis of gestational diabetes	3 (7.5%)	3 (15.0%)	0 (0.0%)	0.23
Current diagnosis of diabetes	3 (7.5%)	3 (15.0%)	0 (0.0%)	0.23
Current diagnosis of chronic hypertension	0 (0.0%)	0 (0.0%)	0 (0.0%)	
Preeclampsia in previous pregnancy	9 (22.5%)	9 (45.0%)	0 (0.0%)	0.001
Gestational age at delivery (weeks)	35.4 (4.6)	31.3 (2.7)	39.5 (1.0)	<0.0001
Birth weight (grams)	2501 (1037)	1690 (782)	3313 (457)	<0.0001
Male infant	15 (37.5%)	5 (25.0%)	10 (50.0%)	0.10
Gestational age at Visit 1	10.9 (2.6)	10.9 (2.8)	10.8 (2.5)	0.92
Gestational age at Visit 2	25.5 (1.0)	25.3 (1.0)	25.6 (1.0)	0.40

^a^p-values calculated with Wilcoxon Rank Sum test, Chi Square test or Fisher Exact test as appropriate. SD = Standard deviation; eoPE = early-onset preeclampsia.

To test the robustness of these measurements, we corrected for the increase in DAO levels at Visit 1 by normalizing our ELISA-based DAO measurements using published DAO activity data. For this purpose we constructed a mean DAO activity curve using non-linear 4PL regression from five different published DAO activity curves^26–30^. The plateau DAO activities were set to 100% (Supplementary Fig. S5). ELISA-based DAO values (Fig. 5a) were then divided by the GW-matched calculated normalization factors and statistically analyzed. This detrending method removed the GW-dependent increase of DAO concentrations at Visit 1. DAO concentrations at Visit 1 from eoPE cases were again significantly decreased when compared to healthy controls with a mean fold difference of 1.6 (p = 0.044) (Fig. 5b). Again, no difference was detected for Visit 2 (p = 0.453). The same samples have been measured twice with comparable results (Supplementary Table S1).

## Discussion

Recent data suggest that EVTs gain access to the maternal blood stream already in the first trimester of pregnancy. These cells not only remodel and colonize spiral arteries but also contact and invade veins and lymphatics^5,31,32^. We therefore reasoned that EVT-derived secreted factors could already be measured in the plasma of pregnant women early in gestation and might be altered in trophoblast-related pathologies. Here we unveil that placenta-derived DAO is exclusively expressed and secreted by EVTs. Interestingly, DAO protein expression is restricted to a subset of EVTs and the percentage of DAO^+^ EVTs is more than doubled when they are in proximity to decidual vessels. These findings may be interpreted in different ways: - EVTs-specific DAO expression could be induced by maternal signals depending on the localization within the maternal uterus; or - DAO^+^ EVTs may represent a unique trophoblast subtype destined to target the decidual vasculature. The latter assumption is supported by the fact that non-uniform DAO expression is already found at the distal end of anchoring villi where environmental influences should be the same for all cells and by the fact that pure cultures of HLA-G^+^ EVTs also show heterogeneous expression of DAO *in vitro* independent of uterine signals. Consequently, comparative and functional analyzes of HLA-G^+^/DAO^−^ and HLA-G^+^/DAO^+^ EVTs are a current focus of our laboratory. In addition, we show that DAO^+^ EVTs integrate into the endothelium of arterial and venous vessels as early as GW 6. These observations are supported by our ELISA measurements detecting a significant rise of plasma DAO by GW 7. Since spiral arteries are mostly plugged in the first trimester of pregnancy^33^, the early rise of DAO plasma concentrations likely originate from EVTs that invade decidual veins. Secreted DAO molecules could alternatively simply diffuse into maternal lymphatic capillaries.

In Homo sapiens DAO is the only extracellular enzyme able to oxidize and inactivate histamine^34^. It has been postulated for decades that pregnancy-specific DAO functions as a safeguard against adverse histamine^12^ since histamine has been shown to induce contractions in human myometrial biopsies^35^ and spontaneous abortions when injected into pregnant animals^36^. However, no data have been published to prove that physiological DAO concentrations during pregnancy are able to efficiently degrade histamine^37^. Interestingly, our data show that pregnancy serum rapidly degrades histamine at pathophysiologically relevant concentrations to non-symptom causing levels (<2 ng/ml). There are likely various sources for undesired histamine during pregnancy. First, histamine might be released at the maternal-fetal interface by adversely activated mast cells. Mast cells, which store large amounts of histamine, accumulate during pregnancy in uterine tissues^38^. Indeed, there is evidence for adverse activation of uterine mast cells during pregnancy^38,39^. Moreover, various data suggest uterine production of histamine^16^ as it is believed to play an important role during implantation^40^, embryogenesis^41^ as well as decidualization^42^. Along these lines, secretion of DAO by EVTs could be beneficial to balance locally produced histamine levels and to prevent excessive histamine efflux into the maternal circulation. This suggests that pregnancy-associated DAO may constitute an efficient safeguard against deleterious effects of circulating as well as locally produced histamine.

Some placenta-derived markers have already been proposed to predict PE in the first trimester of pregnancy including placental growth factor and pregnancy-associated plasma protein A (PAPP-A)^43,44^. For instance, first trimester PAPP-A levels were found to be decreased (1.2 fold) in women who developed PE later in pregnancy by screening a large prospectively collected cohort^44^. In this study we show that DAO levels are significantly lower in the first trimester of pregnancy in women who developed eoPE compared to healthy controls. Since our data suggest that DAO is exclusively derived from EVTs, our results support the theory of incomplete vascular remodeling as causative event in the pathogenesis of eoPE. The lower DAO concentrations in eoPE could also be explained by a decreased half-life of DAO in the maternal circulation or by a reduced diffusion rate of DAO after secretion from EVTs. However, since no difference in DAO levels was observed in eoPE plasma later on in gestation (GW23-27) a delay in EVT-mediated vascular invasion seems more conclusive. This delay may be compensated later in gestation by enhanced trophoblast invasion. Indeed, a previous study suggests that defects in arterial remodeling upon depletion of uterine natural killer cells can be compensated by hypoxia-driven augmented rat trophoblast giant cell invasion^45^. Similarly, PAPP-A levels in women who later on develop PE are decreased in the first trimester of pregnancy, show no difference to healthy controls in the second trimester and even increase in the third trimester when the disease is established^44^. This again suggests compensatory mechanisms and highlights the importance of measuring putative biomarkers for oePE in prospective cohorts.

### Limitations of the study

Although, DAO + EVTs infiltrate decidual vessels and secrete large amounts of the enzyme we cannot exclude that maternal organs such as the kidney or intestine also contribute to the rise in DAO plasma levels during pregnancy. However, there are various reasons to propose EVTs as the main source for the pregnancy-associated rise in circulating DAO levels. First, DAO serum activity abruptly declines after delivery to normal levels^28^; second, DAO^+^ EVTs preferentially locate to decidual vessels and thus gain direct access to the maternal circulation; third, isolated EVTs secret 100-fold more DAO into the supernatant than intestinal cells. Last, DAO activity is at least 25-fold higher in the retroplacental intervillous space when compared to the maternal circulation suggesting its origin at the fetal maternal interface^46^. Moreover, due to restricted availability of suitable prospective biobanks, reduced DAO levels in eoPE have been demonstrated in a small cohort only. Therefore, it would be interesting to test DAO concentrations in larger, multi-centered cohorts. Such an approach would also allow to test whether maternal demographic characteristics and/or medical histories influence the predictive power of DAO.

In summary, this study finds no evidence to support the existence of DAO-producing decidual cells but reveals that bioactive DAO is specifically secreted into the maternal circulation by invasive EVTs. DAO-containing serum from pregnant women rapidly inactivates pathophysiological histamine concentrations; we therefore suggest that placental EVTs may protect the mother and the fetus from excessive endogenous or exogenous histamine. DAO antigen levels represent the first EVT-derived factor with the potential to stratify high risk patients in the first trimester of pregnancy. Finally, it will be interesting to investigate, whether other yet to be identified EVT-derived factors or known biomarkers for eoPE show similar or additive potential to predict eoPE early in gestation.

## Methods

### Tissue collection

Placental and decidual tissues (gestational week (GW) 6–12, n = 72) were obtained from legal, elective pregnancy terminations (approval number: EK084/2009). Gestational age was determined by ultrasound and vacuum suction was done after patients were locally anaesthetized. Third trimester placental specimen were obtained from caesarean sections (GW 37–39, n = 4) (approval number: EK619/2006). Postpartum hysterectomies (n = 3) from women diagnosed with placenta accreta were retrieved from the archive of the Clinical Institute of Pathology, Medical University of Vienna (MUV) (approval number: EK530/2009). Utilization of tissues and all experimental procedures were approved by the local Ethics Committee of the MUV. Methods were carried out in accordance with the approved guidelines. Written informed consent was obtained from all patients.

### Biological samples

EDTA plasma samples (GW 6–14, n = 100,) were obtained from women undergoing legal, elective pregnancy terminations at the GynMed Clinic (Vienna) (approval number: EK1125/2014). Four pregnancy sera (3^rd^ trimester of pregnancy) used for determination of the histamine degradation rate were collected as part of a clinical study conducted at the Department of Obstetrics and Gynaecology (MUV) (approval number: EK1666/2012). These women were included in the normal pregnancy control group. To determine the basal DAO concentration in non-pregnant individuals, 32 sera of healthy volunteers were collected as part of a Phase 1 study before administration of study medication at the Department of Clinical Pharmacology (MUV) (approval number: EK954/2010). These studies were approved by the Ethics Committee of the MUV. Early-onset PE and matched control plasma samples were obtained from the Brigham and Women’s Hospital (Boston, MA). The controls were matched to all parameters listed in Table 1 except preeclampsia in previous pregnancy, gestational age at delivery and birth weight. These are the only parameters significantly different between eoPE and control cases. The samples have been collected as part of the LIFECODES prospective birth cohort study^47^ and were provided to us via the Global Pregnancy Collaboration (CoLab) (approval number: 2009P000810/PHS). All subjects signed an informed consent form before blood withdrawal.

### Isolation and cultivation of primary trophoblasts and decidual cells

Cytotrophoblasts (CTBs) were isolated by enzymatic dispersion and Percoll density gradient centrifugation of pooled first trimester placentas (*n* = 2–5 per isolation) as described^48^. CTBs were cultured for up to 72 hours with media change after 24 hours. First trimester human decidual stromal cells were isolated, cultivated and decidualized for 6 days as described^48,49^.

### Isolation and differentiation of purified primary vCTBs

Villous cytotrophoblast (vCTB) cell populations were isolated as recently published (GW 6–9, *n* = 3–6 per isolation)^2^. To obtain vCTBs, cell column trophoblasts and syncytiotrophoblasts were removed and a second digestion step of the remaining tissue was performed as described^2^. Contaminating erythrocytes extravillous trophoblasts (EVTs) were removed as previously described^2^. vCTBs were seeded onto fibronectin-coated dishes at a density of 3.25 × 10^5^ cells per cm^2^. vCTBs were lysed for western blot analysis after 24 and 96 hours.

### Immunofluorescence of paraffin-embedded tissue

Paraffin-embedded first trimester placental and decidual tissues were proceeded as recently published^2^. Incubation with primary and secondary antibodies (Supplementary Table S2) was performed as published^2^. Images were acquired with a fluorescence microscope (Olympus BX50) or with a confocal laser scanning microscope (LEICA, TCS SP8X). Confocal images are depicted as maximum projection of total z-stacks and brightness and contrast were adjusted in a homogenous manner using Leica LAS AF.

### siRNA-mediated gene silencing

Gene silencing using a pool of 4 different siRNAs targeting AOC1 or a non-targeting (ntc) control pool (L-009218-00-0005 and D-001810-10-20 ON-TARGETplus SMARTpools, Dharmacon-Thermo Fisher Scientific) was performed as published^48^.

### Western blotting

Protein extracts were immobilized on PVDF membranes and incubation with primary and secondary antibodies (Supplementary Table S2) was performed as published^2^. Signals were developed using ECL prime detection Kit (GE Healthcare) and visualized with FluorChemQ imaging system (Alpha Innotech). Signal quantification was performed using Image J software.

### Invasion assay

Invasion assays using primary trophoblasts were carried out using fibronectin-coated transwells (Millipore) as published^48^. Five non-overlapping pictures of each membrane representing ∼50% of the overall surface area were investigated (40-fold magnification) and digitally analyzed using ImageJ software.

### DAO activity assay

DAO activity was measured using a chemiluminescence‐based enzyme activity assay^14^ as described^50^. Briefly, DAO released hydrogen peroxide is used to oxidize luminol by horseradish peroxidase. The generated light can be quantified in a luminometer. We used duplicates of 110 µL final volume in white luminescence plates (204003; Porvair; UK). The 110 µL were composed of 50 µL cell culture supernatants and 50 µL luminol solutions from the Amersham ECL Western Blotting Detection Kit (RPN2108, GE Healthcare Bio-Sciences, Austria) and 2 µg/mL horse radish peroxidase (P6782, Sigma Aldrich). The reaction was started with the addition of 10 µL putrescine (P5780, Sigma Aldrich). The RLU (relative light units) were measured for 180 minutes at 30 °C in 5 minutes intervals. All measurements were performed in a Victor2TM 1420 Microtiter Plate Reader (Perkin Elmer, Austria). We did not determine the minimum detectable DAO concentration in cell culture supernatants, but 20 ng/ml recombinant human DAO in PBS with 0.1% purified human serum albumin (Albunorm, Octapharma, Vienna, Austria) can be readily detected using this assay.

### Quantitative PCR

RNA isolation, reverse transcription and qPCR analyzes were performed as described previously^51^ using TaqMan Gene Expression Assays: AOC1 (Hs00175631_m1), HLA-G (Hs00365950_g1) and ADAM12L (Hs 00185774_m1). Signals (ΔCt) were normalized to TATA-box binding protein (TBP) (ABI, 4333769 F).

### DAO ELISA and determination of DAO concentrations

The development and characterization of the DAO ELISA was recently published^13^. Briefly, a cell culture purified monoclonal antibody raised against human DAO isolated from Caco-2 cell supernatant^23^ was coated onto white high protein binding microtiter plates (655074, Greiner bio-one, Austria) at 5 µg/ml in 50 mM carbonate-bicarbonate coating buffer pH 9.6 overnight at 4 °C (C3041, Sigma-Aldrich, Austria). The hybridoma cell line was provided by Prof. Quaroni (Cornell University). 20 µl EDTA plasma was diluted 1 to 5 with LowCross Buffer (1000500, CANDOR bioscience; Germany). Bound human DAO was detected with a polyclonal rabbit IgG serum fraction diluted 1 to 1000. Rabbits were immunized with purified recombinant human DAO^50^ and serum IgGs isolated using standard procedures (Eurogentec Polyclonal Antibody Production Service, Seraing, Belgium). The bound rabbit antibodies were detected with a 1 to 32000 dilution of donkey anti-rabbit IgG HRP-labelled antibodies (SAB3700928, Sigma-Aldrich, Austria) in the presence of 10 µg/ml donkey IgG (ChromPure #017-000-003; Jackson ImmunoResearch). All incubation steps were performed for 50 minutes at room temperature and standard blocking, incubation and washing solutions were used. Bound HRP molecules were quantified with the SuperSignal™ ELISA Pico Chemiluminescent Substrate (37070, Thermo Scientific, Austria) using a standard chemiluminescent reader (Victor2^TM^ 1420 Microtiter Plate Reader (Perkin Elmer, Austria). A standard curve using 0 to 450 ng/ml recombinant human DAO purified and quantified as described^50^ was used to calculate the concentration of DAO in EDTA plasma samples via 4PL non-linear regression. The calculated standard blank 4PL value was subtracted from the samples. The limit of blank (LOB) and detection (LOD) are 0.27 and 0.48 ng/ml respectively using 42 standard curve determinations. The estimated limit of quantification (eLOQ) is 0.70 ng/ml. Using 42 assays performed over several months the mean (SD) inter-assay coefficient of variation (CV) was 12% (3.4%) in the DAO range of 0.6 to 6 ng/ml and 2% (SD 1.5%) between 17 and 450 ng/ml. Using three times 12 identical EDTA plasma samples with 2.3, 4.4 or 24 ng/ml DAO on the same plate the intra-assay CV was 11%, 11% and 5% respectively. The overall CV of the mean of thirteen pregnancy sera measured twice within 6 months and thirteen EDTA samples from the subjects was 5.2%^13^.

We tested two commercially available DAO ELISAs, but the performance was not satisfactory. The first ELISA (E90656Hu; Uscn Life Sciences Inc., Wuhan, China) was not able to detect active recombinant human DAO and DAO in pregnancy samples and was not further evaluated. The second DAO ELISA (K8500; Immundiagnostik AG, Bensheim, Germany) was tested several times and data comparing the performance with our in-house ELISA have been recently published^13^. The main limitations are that K8500 measures 16 ng/ml DAO using just PBS and shows inadequate linearity using pregnancy samples with 100 to 200 ng/ml DAO or recombinant active human DAO. In the manual of K8500 it is shown that the difference in the median between DAO levels in second and third trimester and healthy controls is only twofold, but during pregnancy DAO concentrations and activity increases several hundredfold.

### Histamine degradation assay using LC-MS/MS

Histamine base was purchased from Sigma-Aldrich. D4-histamine was obtained as dihydrochloride from Santa Cruz Biotechnology. Serum samples were analyzed using liquid chromatography (LC) – tandem mass spectrometry (MS/MS) on a Qtrap 5500 system (Sciex) equipped with a TurboIon Source in positive electrospray ionization mode.

A Symbiosis ALIAS chromatographic system (Spark Holland B.V.) was used. 50 μl of sample was mixed with 10 μl internal standard (d4-histamine 50 μg/mL water), precipitated with 250 μl acetonitrile, vortexed and centrifuged at 12.500 × g for 5 min.

Analytes were separated on an Acclaim HILIC-10 (3 μm, 120 Å, 150 × 2.1 mm, Dionex) by gradient elution using acetonitrile/water/100 mM ammonium formate (50:45:5) and acetonitrile/100 mM ammonium formate (95:5) at a flow rate of 0.4 mL/min. Quantification was performed in multiple reaction monitoring mode (MRM). The mass transitions used for histamine were m/z 112.0→95.0 and for the internal standard d4-histamine m/z 116.1→99.2. Calibration curves in plasma were linear over the concentration range of 1–150 ng/mL with a limit of quantification at 0.75 ng/mL. Relative standard deviations for inter-day and intra-day precision were within 10% for low and high quality controls. Bias of accuracy was below 1%. Selectivity, autosampler stability and carry-over were also tested and met the required criteria. The method was validated using the software Valistat 2.0 (Arvecon, Walldorf, Germany). For routine analysis low and high quality controls for sera were analyzed against the calibration curve and all were within the acceptance criteria of 15%.

Histamine degradation speed was determined by incubating sera from women in the third trimester (n = 4) with 100 ng/mL (0.9 µM) histamine base for various time points at 37 °C. DAO concentration was determined with the DAO ELISA. The reactions were stopped by adding a final concentration of 50 µM diminazene aceturate (Sigma, D7770). Plasma samples from non-pregnant individuals or DAO inhibited with 10 µM diminazene, a potent and selective DAO inhibitor with an inhibition constant Ki of 13 nM against recombinant human DAO, are not able to degrade histamine over 120 minutes. Diminazene reduced the DAO activity to less than 5%. The samples were then immediately snap-frozen in liquid nitrogen and stored at −20 °C until measurement of histamine concentration using LC-MS/MS. Half-life calculations (“one way exponential decay model”) were performed using GraphPad Prism Version 6. Samples were only measured once in duplicates and the mean is shown.

### Statistics

#### ELISA measurements in eoPE and healthy control cohort

To take into account the non-linear increase of DAO values in the first trimester with gestational age and their right-skewed distribution, DAO values were log-transformed using the natural logarithm before entering regression analysis. A multivariable linear model was then fit, explaining log-DAO in the first trimester by the grouping factor patient versus control and by the continuous co-variable gestational age. Model diagnostics supported the assumption of linearity, variance homogeneity and approximately normally distributed errors for this model on the log-scale. The model was used to test the null hypotheses of no association between DAO levels and group while adjusting for the effect of gestational age. The log-linear model further provided an estimate for the fold change of median DAO values between patients and healthy subjects of the same gestational age. A linear model, explaining raw DAO values through group and gestational age, was appropriate for the analysis of DAO values from the second trimester.

### Normalization of DAO concentrations

We used 5 published DAO mean activity curves^26–30^ and calculated DAO activities over the published time frame setting the plateau activity at 100% (Supplementary Fig. S1). To our knowledge these data are the best available measurements using the state of the art DAO activity assay. The manually extracted activity and corresponding gestational age values were used in 4PL non-linear regression analysis to obtain the 4PL parameters for each DAO activity curve. These parameters were used to calculate the relative percent DAO activity from GW 2 to GW 28 in 2 week intervals. The mean values of these 5 curves were again used for 4PL non-linear regression to obtain the 4PL parameters of the mean curve (adjusted R^2^ = 0.999). For each DAO concentration measured with the DAO ELISA a corresponding normalization factor at the same gestation age was calculated. The ELISA-based DAO concentration was divided by this factor and the two sample populations were compared with a t-test with unknown variances. For example, the ELISA-based DAO concentration of one sample at GW 11.1 is 15.1 ng/ml. The corresponding normalization factor at GW 11.1 is 25.9. The ratio of these two numbers 0.58 was used for the t-test. There is no difference comparing the two means of the normalization factors. The mean of the normalization factor in the eoPE cases (n = 20) is 26.3 (arbitrary units) versus 27.4 in the matched controls (n = 20; p = 0.84).

### General

Gaussian distribution and equality of variances were examined with Kolmogorov–Smirnov and Levene test, respectively. Statistical analysis of data between two means was performed with Student’s t test or Mann–Whitney U test using SPSS 14. Comparisons of multiple groups were evaluated with one-way ANOVA and post hoc tests (Tukey when equal variances were assumed, Games-Howell when equal variances were not assumed). A p value of <0.05 was considered statistically significant.

### Data Availability

The authors declare that the data supporting the findings of this study are available within the article and its Supplementary material or from the corresponding authors on request.

## Electronic supplementary material

Supplementary Information
