# Supplementary material for: Pregnancy-associated diamine oxidase originates from extravillous trophoblasts and is decreased in early-onset preeclampsia
Source: Sci Rep. 2018 Apr 20;8:6342. doi: 10.1038/s41598-018-24652-0 (PMC5910386; doi:10.1038/s41598-018-24652-0)
Supplement: Supplementary file 1 — Supplementary Information [file 41598_2018_24652_MOESM1_ESM.pdf]

Supplementary Information

Pregnancy-associated diamine oxidase originates from extravillous trophoblasts and is decreased in early-onset preeclampsia

Philipp Velicky, Karin Windsperger, Karin Petroczi, Sophie Pils, Birgit Reiter, Tamara Weiss, Sigrid Vondra, Robin Ristl, Sabine Dekan, Christian Fiala, David E Cantonwine, Thomas F McElrath, Bernd Jilma, Martin Knöfler, Thomas Boehm, Jürgen Pollheimer

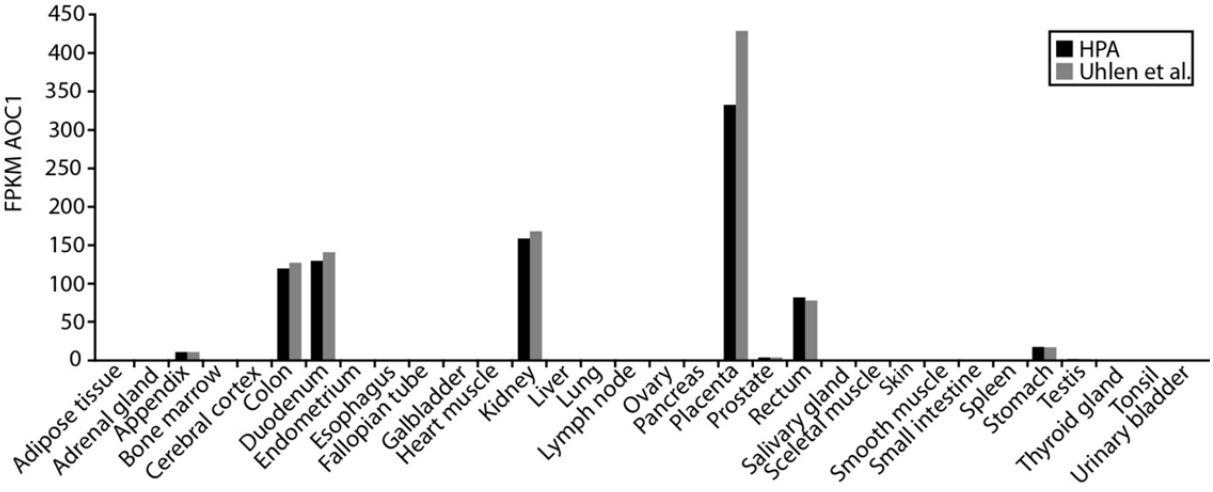

Supplementary Fig. S1. DAO transcript is enriched in the placenta. Levels of corresponding DAO mRNA (shown as fragments per kilobase million, FPKM) in adult organs and the placenta analysed with RNAseq. Source: The Human Protein Atlas portal (www.humanproteinatlas.org) and [22].

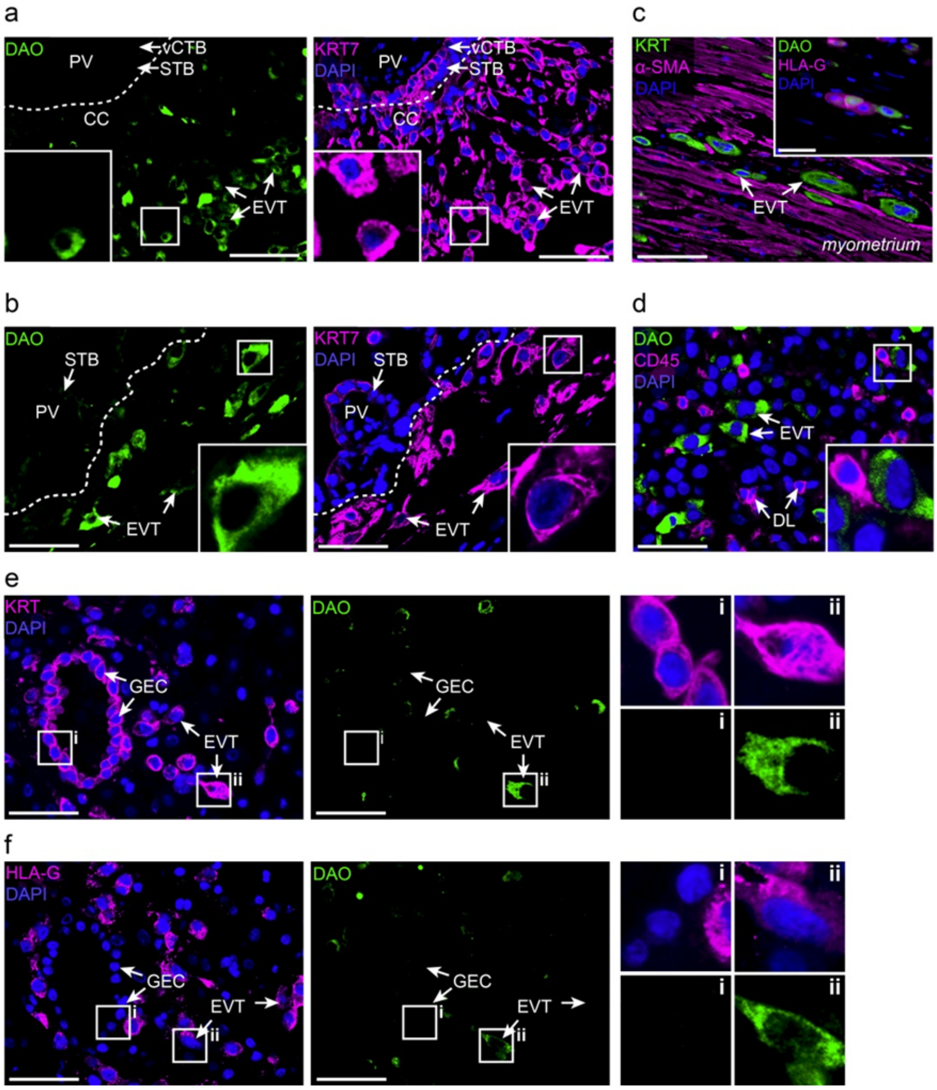

Supplementary Fig. S2. DAO is an EVT-specific factor in the fetal-maternal interface. (**a**) Staining of a first trimester placental tissue (GW 9) section of DAO (green) on the left image and keratin 7 (magenta) and DAPI (blue) on the right image. Dotted line indicates the border between extraembryonic (placental villus) and maternal (decidua) tissue. Insets show digitally zoomed areas indicated by a white rectangle. (**b**) Staining (GW 38) of DAO (green) on the left image and keratin 7 (magenta) and DAPI (blue) on the right image (GW 37 - 39). Dotted line depicts the border between extraembryonic (placental villus) and maternal (decidua) tissue. Insets show digitally zoomed areas indicated by a white rectangle. (Scale bars, 50 μm) (**c**) Staining of myometrial tissue sections obtained of a placenta percreta-conditioned hysterectomy (GW 38) of KRT7 (green), αSMA (magenta) and DAPI (blue) (Scale bar, 100 μm). Inset in the upper right corner shows staining of a serial section of the same tissue of DAO (green), HLA-G (magenta) and DAPI (blue). (Scale bar, 50 μm) (**d**) Staining of a first trimester decidua basalis tissue (GW 10) for DAO (green), CD45 (magenta) and DAPI (blue). Inset shows a digitally zoomed area indicated by a white rectangle. (scale bar, 50 μm) (**e**) Staining of a decidual basalis tissue section (GW 9) for keratin 7 (magenta), DAPI (blue) and DAO (green). The digitally zoomed images (indicated by a white rectangle) demonstrate absence of DAO in keratin 7^+ GECs (i) and an EVT (ii) positive for keratin 7 and DAO. (**f**) A serial section of (**e**) was stained for HLA-G (magenta), DAPI (blue) and DAO (green). The digitally zoomed images confirm the absence of EVT-specific HLA-G in keratin 7^+ GECs (i) and show an EVT (ii) positive for HLA-G and DAO. All images show representative immunofluorescence stainings of 4 or more sections of at least 4 placental or decidua basalis tissues (GW 7-12 and 37-39). CC: cell column, DL: decidual leukocyte, GEC: glandular epithelial cell, PV: placental villous, STB: syncytiotrophoblast.

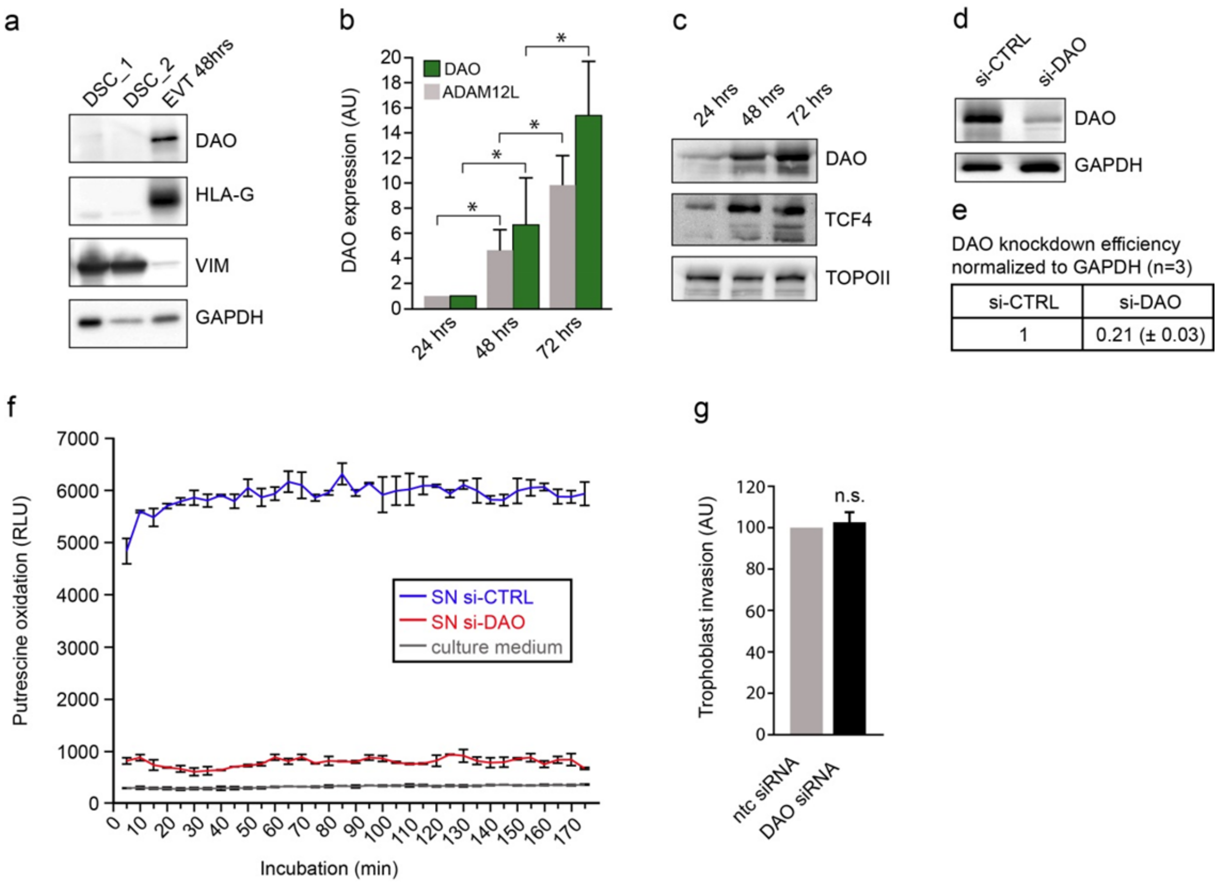

Supplementary Fig. S3. DAO transcription is induced in differentiating CTB cultures and has no influence on trophoblast invasion in vitro. (**a**) Western blot illustrating DAO, HLA-G and VIM in isolated DSCs and differentiated EVTs. GAPDH was used as loading control (n = 3; GW 7-10). (**b**) qPCR analysis of DAO and ADAM12 (upregulated in EVTs) expression in differentiating CTB cultures isolated from first trimester placental tissues (GW 7-11). Each bar represents an average of three independent reactions and three technical replicates. (**c**) Representative western blot analysis for DAO and TCF4 (induced in EVTs) expression in differentiating CTB cultures. Topoisomerase II (TOPOII) served as loading control. (n = 3 independent cultures each isolated from 4 pooled placentas, GW 7-12). (**d**) Western blot analysis of DAO knock down performed in isolated CTBs cultivated for 72 hrs. GAPDH served as loading control. (n = 3 independent cultures each isolated from 4 pooled placentas, GW 7-12). (**e**) Pixel intensities of (**c**) were quantified by using densitometry analysis with ImageJ software. Mean values (± SD) of three independent experiments are shown. (**f**) Luminescence-based DAO activity assay showing putrescine oxidation of supernatants from primary trophoblast cultures treated with anti-*AOC1* (DAO) siRNA or non-targeting control siRNA) (n = 3) (**g**) Invasion assay of in vitro differentiated CTBs in the presence of DAO siRNA or non-targeting control siRNA. Bar graphs display relative values in response to siRNA-mediated knock-down of DAO (green) compared to control (grey) (n = 3). Each invasion assay was performed with pooled CTBs from 4 placentas, GW 7-12. *p < 0.05. Uncropped images of western blots can be found in Supplementary Fig. S7.

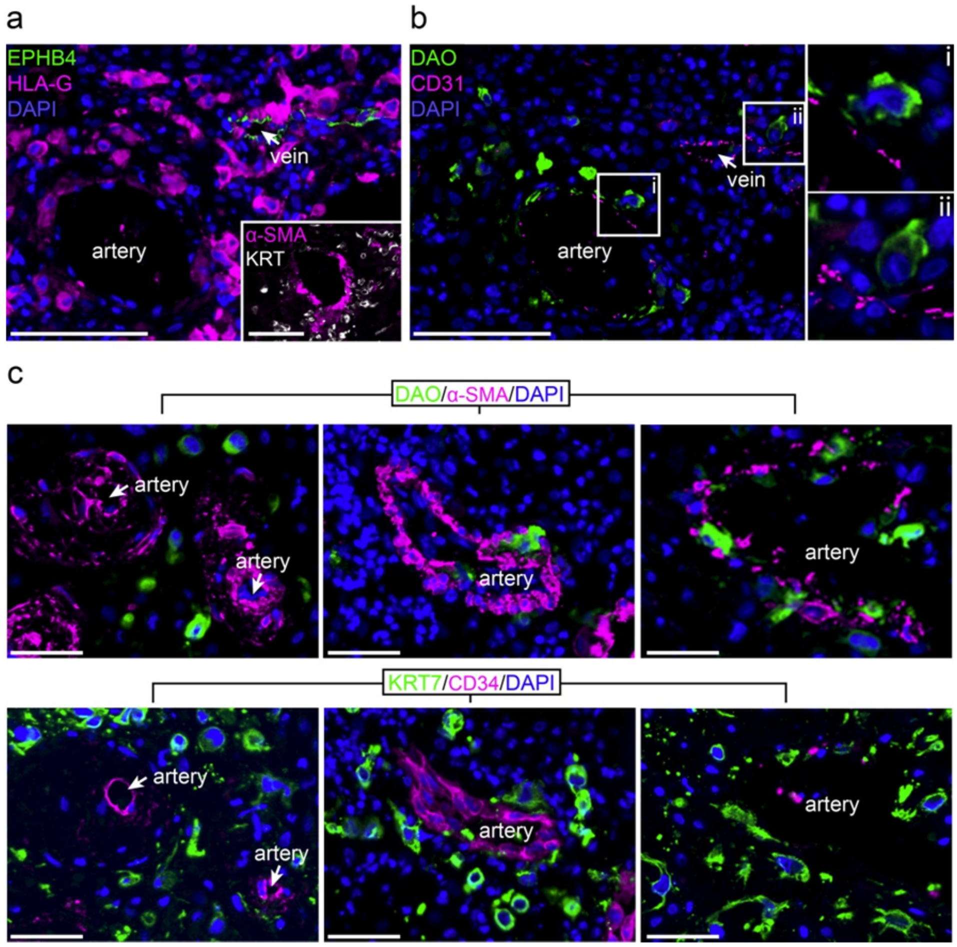

Supplementary Fig. S4. DAO expressing EVTs contact decidual veins and arteries at all stages of remodeling. (**a**) Stainings of decidua basalis tissue (GW 9) sections (3 µm) showing EPHB4 (green), HLA-G (magenta) and DAPI (blue). Inset shows a staining of a serial section for αSMA (magenta) and keratin 7 (grey). (**b**) Serial section (3 µm) of (a) showing DAO (green), CD31 (magenta) and DAPI (blue). The digitally zoomed images (indicated by a white rectangle) demonstrate DAO$^+$ EVTs contacting arterial (i) and venous (ii) vessels. (Scale bar, 100 µm) (**c**) The upper row shows stainings of decidua basalis tissue (GW 10) sections (3 µm) indicating DAO (green), αSMA (magenta) and DAPI (blue). The bottom row shows serial sections of the respective image above indicating KRT7 (green), CD34 (magenta) and DAPI (blue). DAO$^+$ EVTs are found near non-remodeled arteries (left), when breaking the arterial wall (middle) and in remodeled arteries (right). All images show representative immunofluorescence stainings of 4 or more sections of at least 4 placental or decidua basalis tissues (GW 7-12). (Scale bar, 50 µm)

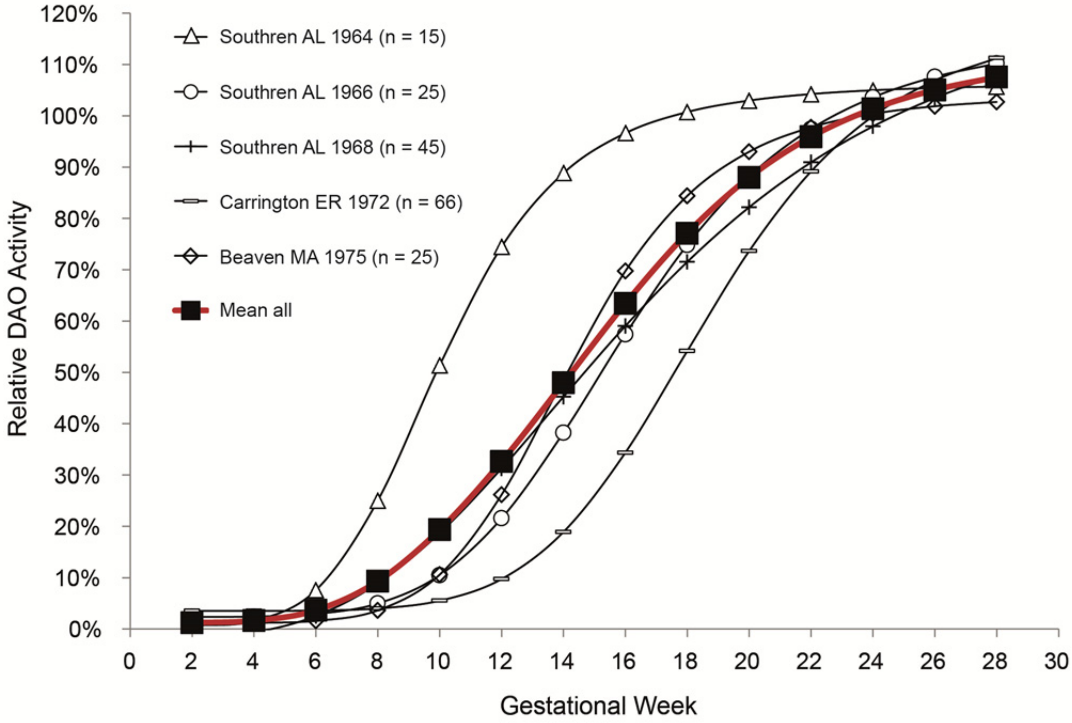

Supplementary Fig. S5. Generation of a mean relative DAO activity curve using a four parameter logistic (4PL) regression of 5 published activity curves**.**

Figure 2 b

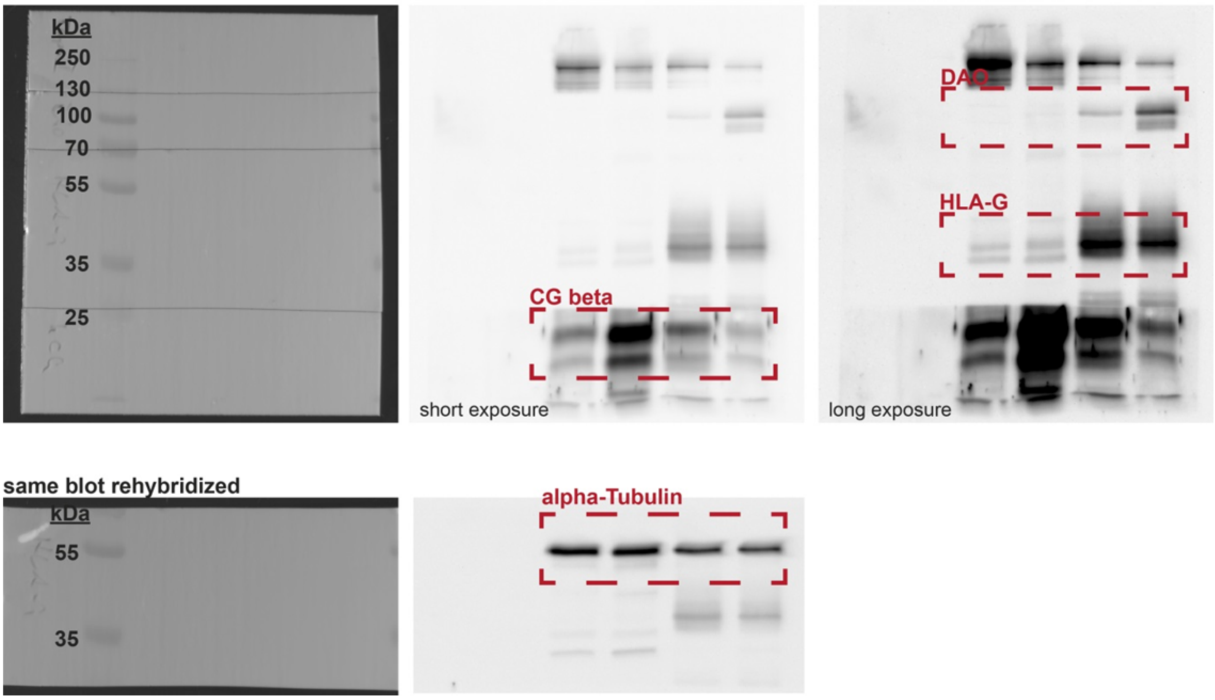

Supplementary Fig. S6. Uncropped western blot images for Figure 2 b. Red, dashed boxes mark the borders of the final cropped image for the indicated protein, respectively. Image on the left indicates positions of molecular weight markers (kDa).

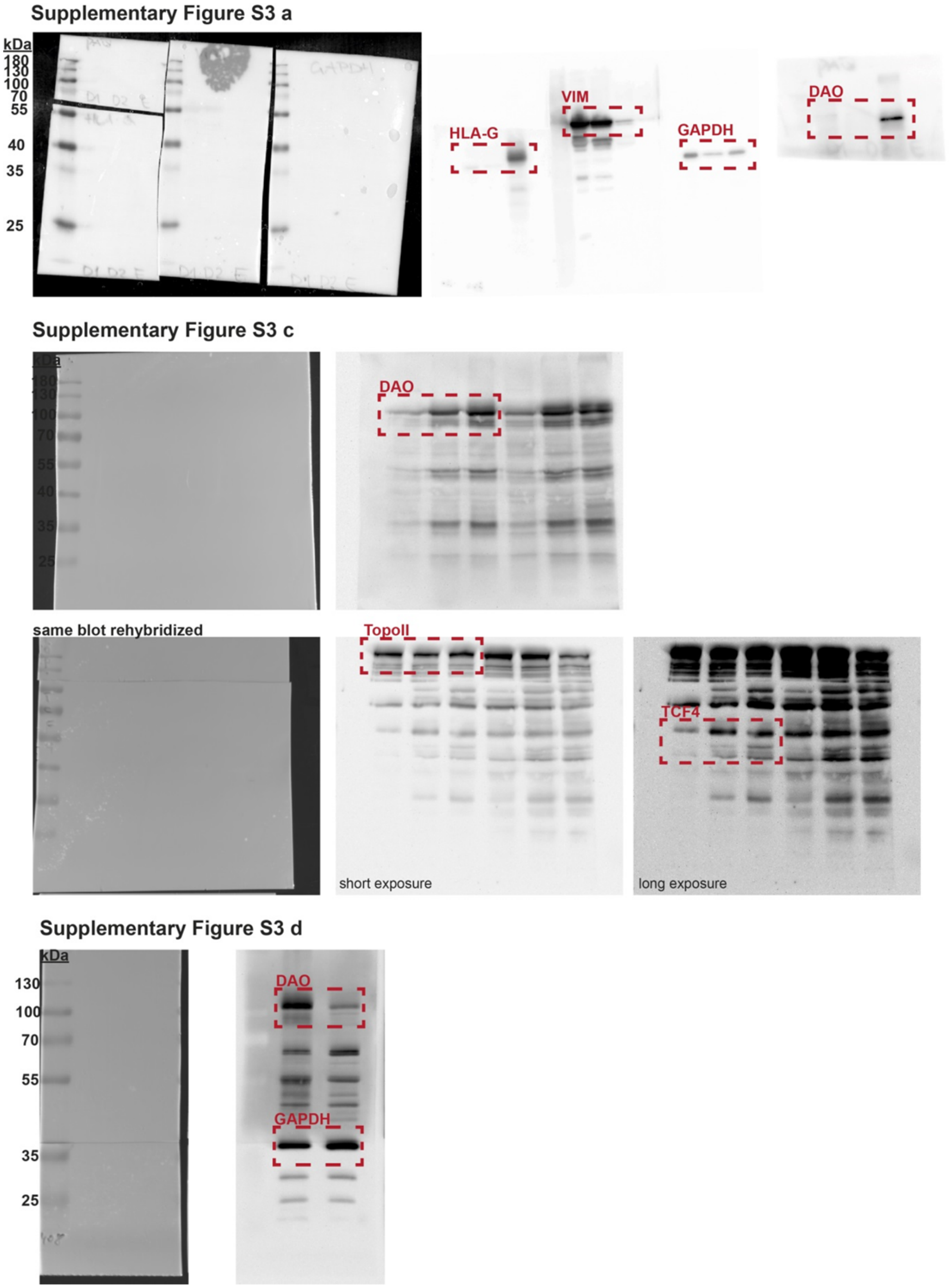

Supplementary Fig. S7. Uncropped western blot images for Supplementary Fig. S3 a, c and S3 d. Red, dashed boxes mark the borders of the final cropped image for the indicated protein, respectively. Images on the left indicate positions of molecular weight markers (kDa).

Supplementary Table S1. DAO plasma concentrations are significantly reduced in the eoPE cohort (First ELISA measurement)

Statistical parameters using DAO ELISA data

	Median ratio	95% CI	p-Value	Regression model
Visit 1 (GW 7-15)	0,55	0,31-0,98	0,043	log-linear
Visit 2 (GW 23-27)			0,368	linear

Statistical parameters using normalized DAO ELISA data

		Mean	95% CI	p-Value	Statistical method
Visit 1 (GW 7-15)	Controls	2,24	1,54-2,94	0,047	t-test
	eoPE	1,35	0,87-1,83		
Visit 2 (GW 23-27)	Controls	2,98	2,19-3,76	0,410	t-test
	eoPE	2,50	1,72-3,29		

CI, confidence interval; GW = Gestational Week; eoPE = early-onset preeclampsia.

Supplementary Table S2. Primary and secondary antibodies.

Antibody	Cat.#	Dilution	Company	Species	Application
HLA-G	NB500-302	1:200	Novus Biologicals	Mouse	IF-P
CD31	Ab9498	1:100	Abcam	Mouse	IF-P
EFNB2	NBP1-84830	1:20	Novus Biologicals	Rabbit	IF-P
EPHB4	14960	1:1000	Cell Signaling	Rabbit	IF-P
α-SMA	M0851	1:50	DAKO	Mouse	IF-P
DAO	HPA031033	1:100	Sigma	Rabbit	IF-P
Cytokeratin (wide-spectrum)	GTX29377	1:100	GeneTex	Rabbit	IF-P
Vimentin	AB5733	1:200	Millipore	chicken	IF-P
Cytokeratin7	M7018	1:200	DAKO	Mouse	IF-P
CD45	M0822	1:100	DAKO	Mouse	IF-P
TCF4	05-511	1:1000	Upstate	Mouse	WB
HLA-G	NB500-302	1:500	Novus Biologicals	Mouse	WB
TOP2B	611492	1:2000	BD Transduction Lab	Mouse	WB
B-HCG	A0231	1:1000	DAKO	Rabbit	WB
DAO	HPA031033	1:1000	Sigma	Rabbit	WB
α-tubulin	CP06	1:5000	Calbiochem	Mouse	WB
GAPDH	14C10	1:5000	Cell Signaling	Rabbit	WB
Anti-mouse 488	A11017	1:1000	Invitrogen	Goat	IF-P
Anti-rabbit 488	A11070	1:1000	Invitrogen	Goat	IF-P
Anti-mouse 568	A11019	1:1000	Invitrogen	Goat	IF-P
Anti-rabbit 568	A11011	1:1000	Invitrogen	Goat	IF-P
Anti-chicken 650	SA5-10073	1:1000	Thermo Scientific	Goat	IF-P
Anti-mouse, HRP	7076	1:10000	Cell Signaling	Horse	WB
Anti-rabbit, HRP	7074	1:10000	Cell signaling	Goat	WB

IF-P, Immunofluorescence of paraffin sections; WB, Western blotting
